# *Klebsiella granulomatis* infection in a patient with human immunodeficiency virus infection

**DOI:** 10.1590/0037-8682-0436-2020

**Published:** 2021-03-08

**Authors:** Max Roberto Batista Araújo, Lincoln Oliveira Sant’Anna, Louisy Sanches dos Santos

**Affiliations:** 1 Instituto Hermes Pardini, Núcleo Técnico Operacional, Setor de Microbiologia, Vespasiano, MG, Brasil.; 2 Universidade Estadual do Rio de Janeiro, Faculdade de Ciências Médicas, Departamento de Microbiologia, Imunologia e Parasitologia, Rio de Janeiro, RJ, Brasil.

**FIGURE 1:**
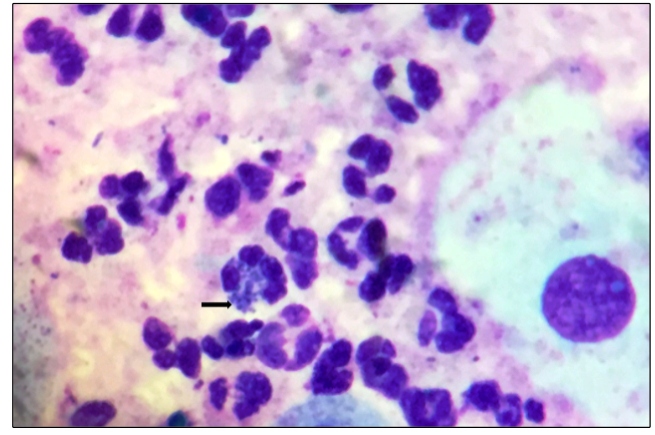
Giemsa staining (original magnification, ×1000) showing Donovan bodies (black arrow).

A 33-year-old man presenting with ulcerated and painless anal lesions was seen by his general practitioner in Belo Horizonte City, Minas Gerais State, Brazil. Screening tests for sexually transmitted infections (STIs) and microscopic examinations of swabs of ulcer material were conducted. Serological examinations gave positive results for human immunodeficiency virus (HIV-1), herpes simplex virus (HSV-1/2), *Treponema pallidum*, and *Chlamydia trachomatis* infections. A microscopic analysis by Giemsa staining showed negative results for Tzank or *Haemophilus ducreyi*; however, it showed Donovan bodies that are characteristic of donovanosis (Figure 1). 

The global incidence of STIs exceeds millions of cases per year, mostly affecting people aged 15-49 years. In tropical and developing areas, donovanosis, also known as granuloma inguinale, is an endemic genital ulcerative disease frequently associated with sexual transmission^1^ ^-^ ^3^. 

Donovanosis is caused by *Klebsiella granulomatis,* an intracellular gram-negative bacterium. Clinically, it manifests as painless, slowly progressive ulcerative lesions in the genitals or perineum without regional lymphadenopathy. The infection can extend to the pelvis or disseminate to the intra-abdominal organs, bones, and mouth. Additionally, secondary bacterial infections in the lesions and co-infections with other sexually transmitted pathogens can be observed^2^ ^-^ ^3^.

*K. granulomatis* is an extremely fastidious organism that is difficult to isolate in artificial culture media. Therefore, the laboratory diagnosis of donovanosis is based on the microscopic visualization of Donovan bodies, dark-staining encapsulated bacteria inside macrophages measuring between 0.6 and 2.5 µm in size^2^ ^-^ ^3^.

Persons with both granuloma inguinale and HIV infection should receive the same regimens as those without HIV infections^2^. 
